# Multimorbidity disease clusters are associated with venous thromboembolism: an extended cross-sectional national study

**DOI:** 10.1007/s11239-024-02987-y

**Published:** 2024-04-27

**Authors:** Jonatan Ahrén, MirNabi Pirouzifard, Björn Holmquist, Jan Sundquist, Kristina Sundquist, Bengt Zöller

**Affiliations:** 1 Center for Primary Health Care Research, Lund University/Region Skåne, Malmö, Sweden; 2 University Clinic Primary Care Skåne, Region Skåne, Sweden; 3 Department of Statistics, Lund University, Lund, Sweden

**Keywords:** Venous Thromboembolism, Multimorbidity, Epidemiology, Medicine, Public health, Genetics medical

## Abstract

Multimorbidity, i.e., two or more non-communicable diseases (NCDs), is an escalating challenge for society. Venous thromboembolism (VTE) is a common cardiovascular disease and it is unknown which multimorbidity clusters associates with VTE. Our aim was to examine the association between different common disease clusters of multimorbidity and VTE. The study is an extended (1997–2015) cross-sectional Swedish study using the National Patient Register and the Multigeneration Register. A total of 2,694,442 Swedish-born individuals were included in the study. Multimorbidity was defined by 45 NCDs. A principal component analysis (PCA) identified multimorbidity disease clusters. Odds ratios (OR) for VTE were calculated for the different multimorbidity disease clusters. There were 16% (n = 440,742) of multimorbid individuals in the study population. Forty-four of the individual 45 NCDs were associated with VTE. The PCA analysis identified nine multimorbidity disease clusters, F1-F9. Seven of these multimorbidity clusters were associated with VTE. The adjusted OR for VTE in the multimorbid patients was for the first three clusters: F1 (cardiometabolic diseases) 3.44 (95%CI 3.24–3.65), F2 (mental disorders) 2.25 (95%CI 2.14–2.37) and F3 (digestive system diseases) 4.35 (95%CI 3.63–5.22). There was an association between multimorbidity severity and OR for VTE. For instance, the occurrence of at least five diseases was in F1 and F2 associated with ORs for VTE: 8.17 (95%CI 6.32–10.55) and 6.31 (95%CI 4.34–9.17), respectively. In this nationwide study we have shown a strong association between VTE and different multimorbidity disease clusters that might be useful for VTE prediction.

**Graphical abstract:**

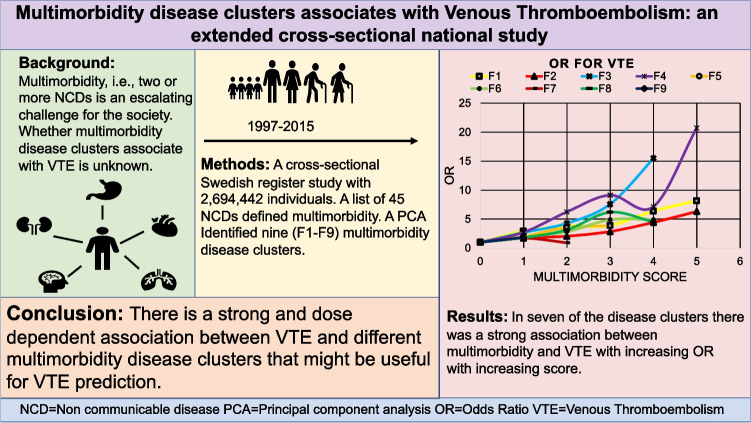

**Supplementary Information:**

The online version contains supplementary material available at 10.1007/s11239-024-02987-y.

## Introduction

Multimorbidity, i.e. two or more non-communicable diseases (NCDs), is a global burden and a challenge for healthcare systems all over the world [1, 2]. With advances in medicine, standards of living and preventive achievement, patients with multimorbidity are more common and live longer [3]. Multimorbidity does not only affect the elderly [4]. It has been reported in a Scottish cross-sectional study to have a prevalence of 23.2% and was common in individuals > 65 years of age [3]. Apart from age, multimorbidity is associated with female sex, low socioeconomic status, smoking, hypertension, high body mass index, and physical inactivity [5]. There is also a genetic inheritance in multimorbidity and, furthermore, multimorbidity aggregates in families [6, 7].

It has been shown in a Swedish nationwide study that multimorbidity defined by 45 NCDs is associated with venous thromboembolism (VTE) [8]. This association was stronger with increased multimorbidity severity, i.e., increased numbers of diseases[8]. VTE is a common and potentially lethal disease and is the third most common cardiovascular disease [9–12]. The incidence of VTE increases with age with an incidence of 0.001% in children and 1% per year in patients above 70 years of age [13]. The outcome of VTE, which comprises pulmonary embolism (PE) and deep vein thrombosis (DVT), may vary from acute death in PE patients (1–2%) to post-thrombotic syndrome (20%) in DVT patients [14, 15]. The mortality rate for VTE is around 5% in 90 days [9]. Risk factors for VTE can be either genetic or acquired. The most salient genetic risk factors are the classic thrombophilia and, among the acquired, it is age [14, 16–19]. Trauma, surgery, malignancy, autoimmune disorders, immobilization, pregnancy, puerperium and oral contraception are also known as acquired risk factors [10, 11, 20-22].

Only one study of multimorbidity and VTE has been published and it is unknown how different disease clusters are associated with VTE [8]. Diseases do not cluster randomly and genetic factors have been suggested to be involved [6, 7]. An initial principal component analysis (PCA) followed by a factor analysis with a principal factor method and an oblique promax rotation is one of several methods to identify disease clusters [7]. These disease clusters reflect the most important multimorbidity clusters and may help to better predict VTE. The dose-graded association with multimorbidity suggests that analysis of the diseases separately may not reflect the total impact on VTE risk [8].

To further investigate the association between multimorbidity severity and VTE, we proceeded to investigate how different disease clusters of NCDs associate with VTE [7]. To date there is no consensus on which diagnoses should be included in the research on multimorbidity [22]. Modified after Barnett et al., we have used a list of 45 common and clinically relevant diagnoses to define multimorbidity and to identify and study different disease clusters [3, 7, 8]. VTE has previously been shown to associate with some of the 45 studied NCDs [3, 8, 23–42]. We hypothesized that certain multimorbidity clusters are more strongly associated with VTE and that the strength of the association differs between the clusters. In this study, we therefore aimed to determine how VTE is associated with different disease clusters of multimorbidity. Several Swedish national registers were used and linked to study the association between different disease clusters and VTE [7, 9, 43–48].

## Material and methods

The methodology used has been described in detail [7, 8]. For clarity a short summary is presented.

### Registers

This study used several nationwide registers: the Swedish Cause of Death Register; the Total Population Register including data on education, migration, marital status and death date; the National Patient Register (NPR) including data on hospital discharge diagnoses between 1964 and 2015 with nationwide coverage from 1987, and outpatient hospital diagnoses between 2001 and 2015. The Swedish Multigeneration registers that hold data on familial relationships were also used [43–48]. To link the databases the Swedish personal identity number (PIN) was used by Statistics Sweden. To protect people’s integrity the PIN was replaced with a serial number and pseudonymized by Statistics Sweden. The Swedish registers have a high degree of coverage and have been previously investigated for their validity [43–48]. The registers were provided by the National Board of Health and Welfare and Statistics Sweden.

### Study design

A cross-sectional nationwide study between 1997 and 2015 [7, 8]. To increase the coverage of the registers, and to use the international classification of disease (ICD) 10 codes, the time was set between 1997 to 2015. To increase the certainty of the PIN only individuals born in Sweden were included and the dataset contains Swedish-born individuals [7, 8, 44]. Individuals who had emigrated or died before 1997 or emigrated before the age of 17 were excluded.

### Definition of venous thromboembolism

The WHO ICD, Tenth Revision (ICD-10) was used to classify cases of VTE. ICD codes: ICD-10: I80, (DVT) (not I80.0) and I26 (PE) were used to define VTE in the main and supplementary diagnoses in the hospital discharge registers between 1997 and 2015 [8]. In the outpatient care register between 2001 and 2015, and in the cause of death register, the same codes were used. The validity of diagnoses in the NPR has previously been s﻿hown to be high (positive predictive value > 85%) for many diagnoses [45]. In the NPR the general validity of diagnosis is 85–95% [45]. Diagnosis of VTE has a high validity of 90–95% [13, 49].

### Multimorbidity severity score

To date there is no consensus regarding which NCDs should be investigated when studying multimorbidity [22]. We used a list of 45 diagnoses that are common, clinically important, contributing to disease burden, modified after Barnet et al., and have previously been published (Table 1) [3, 7, 8]. Multimorbidity was defined as having two or more NCDs [3, 7, 8]. A cumulative multimorbidity score was constructed and defined by 0, 1, 2, 3, 4 or 5 or more NCDs [7, 8].

**Table 1:** Odds ratio (OR) for VTE (venous thromboembolism) and multimorbidity (score ≥ 2) based on 45 diseases and stratified for nine different multimorbidity disease clusters (F1-F9) [7, 8]. Odds Ratios (ORs) with 95% confidence interval (CI) for multimorbidity. Reference with one or no diseases (score = 0 or 1). Total observations n = 2,694,442

	OR (95% CI)			
	No VTE	VTE	Model 1	Model 2
F1	33,815	1312	6.94 (6.55–7.35)	3.44 (3.24–3.65)
F2	125,668	1802	2.56 (2.44–2.69)	2.25 (2.14–2.37)
F3	2973	126	7.10 (5.94–8.49)	4.35 (3.63–5.22)
F4	9674	482	8.51 (7.76–9.34)	5.65 (5.14–6.21)
F5	5708	195	5.74 (4.97–6.63)	3.32 (2.87–3.84)
F6	14,997	506	5.76 (5.27–6.31)	2.56 (2.34–2.80)
F7	249	2	1.34 (0.33–5.38)	0.90 (0.22–3.65)
F8	57,067	672	2.00 (1.85–2.16)	3.08 (2.85–3.33)
F9	6	0	NA	NA

Model 1 is a crude model (univariable). Model 2 is an adjusted model (multivariable), with adjustments for year of birth, region at birth, sex and educational attainment. *Previously published based on 45 diseases [7, 8]. Nine different disease clusters (F1-F9) as previously described  [7]

OR = odds ratio; NA = not applicable due to few cases; F1 = hypertension, heart failure, coronary heart disease, diabetes, obesity, atrial fibrillation, gout, atherosclerosis, and renal disease;F2 = affective disorders, anxiety, psychoactive substance misuse, alcohol misuse disorders, anorexia or bulimia, and schizophrenia disorders; F3 = inflammatory bowel disease, liver disease, pancreatic disease, and ulcers; F4 = epilepsy, blindness and poor vision, cerebrovascular disease, cancer, and impaired or hearing loss; F5 = connective tissue disease, osteoporosis, thyroid disorders, and psoriasis; F6 = prostate disease, arthrosis, painful back condition, diverticular disease of intestine, and chronic sinusitis; F7 = bronchiectasis, Parkinson’s disease, glaucoma, learning disability, and irritable bowel syndrome; F8 = asthma, dermatitis and eczema, constipation, chronic obstructive pulmonary disease, and migraine; and F9 = multiple sclerosis and dementia

### Statistical analysis

Logistic regression was used to investigate the association between VTE and the NCDs comprising multimorbidity [7, 8]. As previously described in detail, a principal component analysis (PCA) followed by a factor analysis was used to identify nine disease clusters (F1-F9) among the NCDs at the individual patient level (Table 1). PCA is a statistical method for exploratory studies to identify clusters [7, 50, 51], see supplement statistical section for detailed information. The association between different disease clusters (F1-F9) and VTE are presented as Odds Ratios (ORs) with 95% confidence intervals (CIs). Adjustments in the logistic regression models were for year of birth, region at birth, level of education, and sex. Statistical significance was set to p < 0.05, all tests were two-tailed. Data were analyzed using STATA V.16.1 (StataCorp LLC, College Station, TX, USA).

### Ethical considerations

The current study was based on secondary data and the use was approved by the relevant ethical authorities (Dnr 2012/795 and later amendments).

## Results

### Multimorbidity and VTE

The study population has previously been described in detail [7, 8]. In summary, the population in the study consisted of 2,694,442 unique individuals where 49% were females [7, 8]. The mean age at the end of the study was 32 years [8]. During the study period between 1997 and 2015, 16% (n = 440,742) were multimorbid [7, 8]. Of the individuals with multimorbidity, 253,931 (57.6%) were females [7, 8]. The proportion of females increased with increasing multimorbidity scores with 63.2% of the individuals with a score of ≥ 5 being female [7, 8]. Multimorbidity increased with lower levels of education and with older age [7, 8]. There was a total number of 16,099 VTE cases diagnosed during the study time, 0.6% of the study population [8]. Among the multimorbid individuals, 1.6% had a VTE diagnosis compared to 0.4% without multimorbidity [8]. There was a total of 6983 diagnosed with VTE in the multimorbid group of 433,759 individuals (1.6%) [8].

### VTE risk in relation to the individual diseases comprising the multimorbidity index

In Supplementary Table S1 the association between the 45 individuals NCDs and VTE are described. Strongest associations were found for atherosclerosis (OR = 7.66, 95%CI 7.02–8.35), heart failure (OR = 5.02, 95%CI 4.47–5.63), bronchiectasis (OR = 4.309, 95%CI 2.77–6.69) and cancer (OR = 3.76, 95%CI 3.57–3.95).

### VTE risk in relation to nine different multimorbidity disease clusters

We determined multimorbidity among nine previously described disease clusters (F1-F9) and VTE (Table 1) [7]. In seven of the disease clusters there was a strong association between multimorbidity and VTE compared with no or one disease (Table 1). Only for the disease clusters F7 and F9, there were no associations between VTE and multimorbidity. The disease clusters with the highest adjusted OR in the adjusted model were the F4 and F3 clusters. The adjusted OR for the F4 cluster was 5.65 (95%CI 5.14–6.21) and the adjusted OR for the F3 cluster was 4.35 (95%CI 3.63–5.22). The two most important disease clusters with the highest eigenvalue were F1 and F2; they had adjusted ORs of 3.44 (3.24–3.65) and 2.25 (2.14–2.37) for VTE, respectively, Supplementary Figure S1.

### VTE risk in relation to scores for nine different multimorbidity disease clusters

The OR for one disease compared with no disease was increased in all nine multimorbidity disease clusters (F1-F9) (Table 2). The OR for two diseases compared with no disease was increased in seven multimorbidity disease cluster groups F1-F6 and F8 but not for F7 and F9 (Tables 2).

**Table 2:** Odds ratio for VTE (venous thromboembolism) for disease clusters F1-F9 according to multimorbidity score (0 to ≥ 5). Odds Ratios (ORs) with 95% confidence interval (CI). Reference with no diseases (score = 0)

	All (n = 2,694,442)		OR (95% CI)	OR (95% CI)
Score (0 to ≥ 5)	No VTE	VTE	Model 1	Model 2
F1 = hypertension, heart failure, coronary heart disease, diabetes, obesity, atrial fibrillation, gout, atherosclerosis, and renal disease (9 diseases)				
F1 Score 0	2,480,568	11,902	1[Reference]	1[Reference]
F1 Score 1	163,960	2885	3.67 (3.52–3.82)	2.92 (2.80–3.04)
F1 Score 2	24,713	842	7.10 (6.61–7.62)	3.80 (3.53–4.08)
F1 Score 3	6729	278	8.61 (7.63–9.72)	3.93 (3.48–4.45)
F1 Score 4	1716	126	15.30 (12.76–18.35)	6.39 (5.32–7.68)
F1 Score ≥ 5	657	66	20.94 (16.25–26.98)	8.17 (6.32–10.55)
F2 = affective disorders, anxiety, psychoactive substance misuse, alcohol misuse disorders, anorexia or bulimia, and schizophrenia disorders (6 diseases)				
F2 Score 0	2,348,952	12,307	1[Reference]	1[Reference]
F2 Score 1	203,723	1990	1.86 (1.78–1.96)	1.73 (1.65–1.82)
F2 Score 2	89,520	1086	2.32 (2.18–2.47)	2.05 (1.93–2.19)
F2 Score 3	27,098	458	3.23 (2.94–3.54)	2.85 (2.59–3.13)
F2 Score 4	8228	229	5.31 (4.65–6.06)	4.43 (3.88–5.07)
F2 Score ≥ 5	822	29	6.73 (4.65–9.76)	6.31 (4.34–9.17)
F3 = inflammatory bowel disease, liver disease, pancreatic disease, and ulcers (4 diseases)				
F3 Score 0	2,628,503	14,948	1[Reference]	1[Reference]
F3 Score 1	46,867	1025	3.85 (3.61–4.10)	2.69 (2.52–2.87)
F3 Score 2	2750	109	6.97 (5.75–8.45)	4.28 (3.53–5.20)
F3 Score 3	209	15	12.66 (7.50–21.37)	7.59 (4.46–12.91)
F3 Score 4	14	2	25.12 (5.71–110.54)	15.5 (3.33–68.88)
F3 Score ≥ 5	NA	NA	NA	NA
F4 = epilepsy, blindness and poor vision, cerebrovascular disease, cancer, and impaired or hearing loss (5 diseases)				
F4 Score 0	2,530,129	12,987	1[Reference]	1[Reference]
F4 Score 1	138,540	2630	3.70 (3.55–3.86)	2.72 (2.60–2.84)
F4 Score 2	8686	415	9.31 (8.42–10.29)	6.24 (5.64–6.91)
F4 Score 3	911	62	13.26 (10.25–17.16)	9.14 (7.03–11.89)
F4 Score 4	73	4	10.68 (3.90–29.21)	7.16 (2.57–19.98)
F4 Score ≥ 5	4	1	48.71 (5.44–435.79)	20.74 (2.10–204.30)
F5 = connective tissue disease, osteoporosis, thyroid disorders, and psoriasis; (4 diseases)				
F5 Score 0	2,561,719	14,197	1[Reference]	1[Reference]
F5 Score 1	110,916	1707	2.78 (2.64–2.92)	1.98 (1.89–2.09)
F5 Score 2	5463	184	6.08 (5.24–7.05)	3.57 (3.07–4.14)
F5 Score 3	243	11	8.17 (4.47–14.96)	3.85 (2.09–7.09)
F5 Score 4	2	0	NA	NA
F5 Score ≥ 5	NA	NA	NA	NA
F6 = prostate disease, arthrosis, painful back condition, diverticular disease of intestine, and chronic sinusitis (5 diseases)				
F6 Score 0	2,460,459	12,471	1[Reference]	1[Reference]
F6 Score 1	202,887	3122	3.04 (2.92–3.16)	1.90 (1.82–1.97)
F6 Score 2	14,277	459	6.34 (5.77–6.97)	2.82 (2.56–3.10)
F6 Score 3	688	45	12.90 (9.54–17.46)	4.90 (3.61–6.64)
F6 Score 4	31	2	12.73 (3.05–53.19)	4.93 (1.17–20.72
F6 Score ≥ 5	1	0	NA	NA
F7 = bronchiectasis, Parkinson’s disease, glaucoma, learning disability, and irritable bowel syndrome (5 diseases)				
F7 Score 0	2,634,690	15,572	1[Reference]	1[Reference]
F7 Score 1	43,403	525	2.05 (1.88–2.23)	1.73 (1.58–1.89)
F7 Score 2	249	2	1.36 (0.34–5.46)	0.92 (0.28–3.71)
F7 Score 3	NA	NA	NA	NA
F7 Score 4	NA	NA	NA	NA
F7 Score ≥ 5	NA	NA	NA	NA
F8 = asthma, dermatitis and eczema, constipation, chronic obstructive pulmonary disease, and migraine (5 diseases)				
F8 Score 0	2,277,416	12,571	1[Reference]	1[Reference]
F8 Score 1	343,860	2856	1.50 (1.44–1.57)	1.80 (1.73–1.88)
F8 Score 2	51,902	570	1.99 (1.83–2.16)	3.15 (2.89–3.43)
F8 Score 3	4902	97	3.58 (2.93–4.39)	6.19 (5.03–7.60)
F8 Score 4	259	5	3.50 (1.44–8.48)	4.51 (1.83–11.10)
F8 Score ≥ 5	4	0	NA	NA
F9 = multiple sclerosis and dementia (2 diseases)				
F9 Score 0	2,672,359	15,988	1[Reference]	1[Reference]
F9 Score 1	5978	111	3.10 (2.57–3.75)	1.86 (1.54–2.24)
F9 Score 2	6	0	NA	NA
F9 Score 3	NA	NA	NA	NA
F9 Score 4	NA	NA	NA	NA
F9 Scpre ≥ 5	NA	NA	NA	NA

OR = odds ratio; NA = not applicable due to few cases. Model 1 is a crude model. Model 2 is adjusted for sex, year of birth, region at birth, and educational attainment

**Fig. 1:**
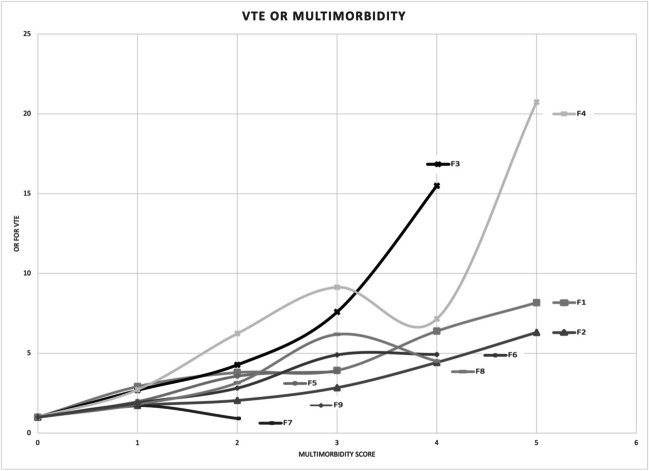
OR for VTE with increasing multimorbidity score for F1-F9, adjusted model (multivariable), with adjustments for sex, year of birth, region at birth, and educational attainment

Figure 1 shows the ORs in relation to multimorbidity score (0 to ≥ 5) for the nine different disease clusters (F1-F9). A pattern with increasing OR with increasing multimorbidity scores could be observed in Fig. 1. For five disease clusters (F1, F2, F3, F5, F6) there was a perfect match between score and OR for VTE (Table 2). In disease cluster F1, OR for a multimorbidity score of two was 3.80 (95%CI 3.53–4.08), for a score of three it was 3.93 (95%CI 3.48–4.45), for a score of four it was 6.39 (95%CI 5.32–7.68), and for a score of five or more it was 8.17 (95% CI 6.32–10.55) in the adjusted model (Table 2). There was a similar increase in OR for disease cluster F2 where an increase in multimorbidity increased the OR for VTE. (Table 2). For F3, F5, and F6 the ORs also increased with the score although there were not enough cases with the highest scores.

For the two disease clusters, F4 and F8, there were increasing ORs for VTE with increasing scores except for the second highest score for F4 and the highest score for F8 (Table 2). The limited number of diseases could explain why the highest applicable score (4) of the disease cluster F8 was associated with an OR of 4.51 (95%CI 1.83–11.10) compared with an OR of 6.19 (95%CI 5.03–7.60) for a score of three (Table 2).

## Discussion

We have previously shown an association between multimorbidity and its severity and VTE [8]. The present study extends these findings to, previously identified, common multimorbidity disease clusters [7]. Our results show a strong association for VTE with seven of nine multimorbidity disease clusters [7]. We also show that higher multimorbidity severity increases the OR for VTE. The strong and graded associations with different common multimorbidity disease clusters are important because different disease-specific multimorbidity clusters could be used instead of individual diseases for prediction of VTE. However, the present study is an extended cross-sectional study, and these results need to be validated in additional follow-up studies. The clustering of the NCDs in the multimorbidity score at the individual patient level according to the PCA and the oblique rotation in the factor analysis has been previously described, but not the association with VTE [7]. In the disease clusters, F7 and F9, there were not enough individuals with multimorbidity and VTE to have definitive results to determine if these disease clusters were associated with VTE. A reason for the strong association between the modified Barnett multimorbidity index is most likely due to that 44 of the 45 included NCDs were all individually associated with VTE (Supplementary Table S1).

The identified clusters by the PCA analysis are in line with previously published literature [7]. The first two disease clusters F1 (cardiometabolic diseases) and F2 (mental health disorders) clusters are often recognized in multimorbidity studies [56]. For example, cardiometabolic diseases in F1 have been described previously to be associated with VTE [30, 31, 39–41]. The diseases in F2 have been shown to be associated with VTE [52–55]. For cluster F3, inflammatory bowel disease, autoimmune liver disease and autoimmune connective tissue diseases are associated with VTE [21, 35, 36]. Moreover, cancer and cerebrovascular diseases in F4 are important risk factors for VTE [20]. However, the present study suggests that the number of diseases an individual is affected by might be of great importance. For VTE it has been shown that many gene variants might be added to a polygenetic risk score (PRS) [57]. If the present study could be replicated in follow-up studies to predict incident VTE it is possible that a multimorbidity risk score for prediction in analogy with a PRS could be constructed and even combined with a PRS. Another question that has not been studied is whether multimorbidity affects the risk of recurrent VTE.

An interesting observation is that all seven multimorbidity disease clusters associated with VTE in the present study have been shown to cluster in families (F1-F6, F8) [8]. The two multimorbidity disease clusters (F7 and F9), which were not significantly associated with VTE in the present study, were not clustering in families either [8]. These two disease groups (F7 and F9) only cluster at an individual level and not at the family level [8]. Some of the associations in the present study between multimorbidity clusters and VTE may therefore have a genetic contribution, which however remains to be studied. An example is obesity in the disease cluster F1 (cardiometabolic diseases). A genome-wide study of body mass index has shown a genetic correlation with VTE [58]. Another example is depression in the F2 cluster (mental health-related disorders) where a recent Mendelian randomization study has suggested a causal association between major depression and VTE [54].

### Strengths and limitations

Due to the study design (extended cross-sectional), it is not possible to allow causal inferences to be drawn. A limitation of the study is that we did not determine the temporal association between VTE and multimorbidity. Of the included NCDs, atrial fibrillation, arterial atherosclerotic diseases, cancer and depression have previously been shown to have a bidirectional association with VTE [39–41, 54, 59, 60]. Due to this limitation in study design, it will be important to determine if any temporal association exists in future studies. Possible limitations are also the limited number of known confounders and the fact that the register data did not include any risk factors related to lifestyle. Therefore, the multivariable analysis was adjusted for sex, year of birth, region at birth, and educational attainment. To make up for the lack of data, education level was used and adjusted for, which has been shown to be lifestyle-related [61]. Educational level is possibly the strongest socioeconomic predictor for good health and is associated with multiple cardiovascular risk factors [61]. A total of 45 diseases are included in the used multimorbidity index [7]. If we adjusted for some of these diseases, different adjustments would be made for the nine different multimorbidity disease clusters that were studied. It would then be hard to directly compare the different disease clusters. This is another limitation of the study. A strength of the study is the high coverage and validity of the Swedish registers [43–49]. The study includes many unique individuals, and the study period was chosen to have high coverage with low missing data. To decrease the risk of inaccurate coding, the ICD-10 codes were used [7, 8]. Since the study individuals are Swedish-born and the geographical location is Sweden the results may not be generalized in countries that are very different from Sweden, and it remains for future studies to investigate. In the study, the patients were relatively young and due to this fact further studies need to be done to examine whether the results can be generalized to an elderly population.

## Conclusion

In this nationwide extended cross-sectional study, we have shown an association between VTE and multimorbidity severity for seven of nine disease clusters. These results suggest that several disease clusters may be used for VTE prediction in clinical settings through knowledge obtained from prospective studies.

### Supplementary Information

Below is the link to the electronic supplementary material.

Supplementary file1 (DOCX 64.1 KB)

## Data Availability

We cannot distribute our data because it originates from registries owned by a third party, namely the Swedish authorities. The rationale for this restriction is to safeguard patient confidentiality and privacy, as public availability of the data could pose a risk. Access to the data is granted to researchers, subject to specific conditions, by the National Board of Health and Welfare and Statistics Sweden. Requests for data usage can be made to the Swedish National Board of Health and Welfare (https://www.socialstyrelsen.se/en/statistics-and-data/statistics/statisticaldatabase/) and Statistics Sweden (https://www.scb.se/en/About-us/contact-us/). To request the same minimal dataset used in this study, applicants must provide a motivation, reference to the current study, and a list of variables. This information should be submitted along with the data request to the Swedish National Board of Health and Welfare and Statistics Sweden using the contact information provided. The authorities will then coordinate the request handling process and conduct a special review.

## Declarations

None declared.
