# Supplementary material for: Multimorbidity disease clusters are associated with venous thromboembolism: an extended cross-sectional national study
Source: J Thromb Thrombolysis. 2024 Apr 27;57(6):898–906. doi: 10.1007/s11239-024-02987-y (PMC11315723; doi:10.1007/s11239-024-02987-y)
Supplement: Supplementary file 1 — Supplementary file1 (DOCX 64.1 KB) [file 11239_2024_2987_MOESM1_ESM.docx]

**Supplement Multimorbidity clusters are associated with venous thromboembolism**

**Statistical section:**

The Principal Component Analysis (PCA) [50,51] was used to identify nine disease clusters that have previously been published [7]. An overview is given below:

PCA is a method used to uncover the primary directions [50,51], which are linear combinations of variables (i.e., diagnoses) representing the predominant co-variation among these diagnoses, as observed in the correlation matrix. These directions, known as eigenvectors, along with their associated eigenvalues describing variances, collectively provide a comprehensive depiction of the correlation structure within the 45 included diagnoses [7]. A reduced set of eigenvectors, characterized by substantial corresponding eigenvalues, effectively replicates the correlation matrix's patterns.

Factor analysis is utilized to interpret the correlation matrix's patterns, separating variations into common factors (referred to as communality) and individual-specific variances unique to each diagnosis, distinct from communality.

To achieve this, one approach involves initiating with PCA decomposition of eigenvectors and then performing an oblique (non-orthogonal) rotation, maximizing the allocation of the correlation pattern into the communality component. This helps capture the influence of potentially correlated common factors.

In this study, we conducted an initial PCA decomposition followed by a factor analysis using the principal factor method and oblique promax rotation on the correlation matrix, which contained tetra-choric correlations among the 45 diagnoses in patients [7]. This approach was used to identify disease clusters. Subsequently, these disease clusters, determined at the individual level through factor analysis, were employed to assess familial risks within these smaller disease clusters [7]. In factor analysis, selecting the appropriate number of factors is crucial. In this study the “elbow” method was used which involves plotting the eigenvalues against the number of factors and identifying the point where the eigenvalues show a significant drop, indicating the optimal number of factors to retain [7]. This approach is based on the principle that factors beyond this point contribute minimally to explaining the variance in data.

**Supplementary Figure S1.** Eigenvalues plot that shows the curve flattening after nine factors [7].


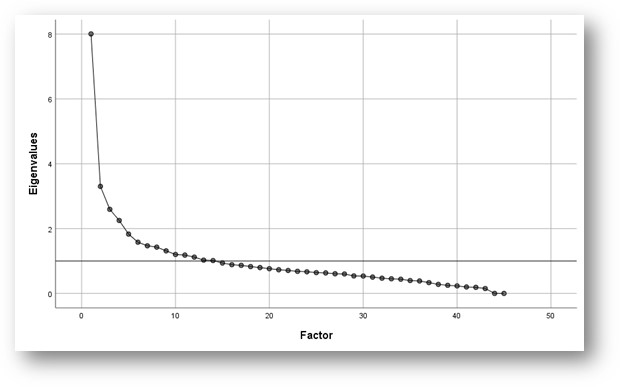


| **Supplementary Table S1.** Odds ratio (OR) for VTE (venous thromboembolism) and multimorbidity (score ≥2) based on 45 diseases [3,7,8]. Total observations n=2694442. Adjusted model (multivariable), with adjustments for year of birth, region at birth, sex, and educational attainment, | | | | |
| --- | --- | --- | --- | --- |
|  | No VTE | VTE | OR | 95% CI |
| Atherosclerosis | 8 156 | 594 | 7.66 | 7.02-8.35 |
| Heart failure | 5 051 | 322 | 5.02 | 4.47-5.63 |
| Bronchiectasis | 534 | 21 | 4.30 | 2.77-6.69 |
| Cancer | 50 518 | 1 931 | 3.76 | 3.57-3.95 |
| Osteoporosis | 3 072 | 132 | 3.69 | 3.09-4.41 |
| Cerebrovascular | 11 064 | 477 | 3.69 | 3.36-4.05 |
| Dementia | 353 | 19 | 3.59 | 2.25-5.71 |
| COPD | 5 231 | 244 | 3.43 | 3.01-3.91 |
| Liver disease | 8 949 | 290 | 3.30 | 2.93-3.71 |
| Renal disease | 51 605 | 977 | 3.28 | 3.07-3.51 |
| Gout | 3 792 | 133 | 3.25 | 2.73-3.88 |
| Psychoactive substance misuse | 40 470 | 819 | 3.19 | 2.96-3.43 |
| Obesity | 69 060 | 1 395 | 2.96 | 2.80-3.3 |
| Connective tissue | 32 719 | 735 | 2.92 | 2.70-3.14 |
| Epilepsy | 29 828 | 524 | 2.87 | 2.63-3.14 |
| IBD | 27 720 | 565 | 2.82 | 2.59-3.07 |
| Atrial fibrillation | 10 202 | 318 | 2.67 | 2.38-2.99 |
| Pancreas | 8 138 | 208 | 2.66 | 2.32-3.06 |
| Constipation | 62 668 | 573 | 2.62 | 2.41-2.85 |
| Ulcer | 8 243 | 233 | 2.57 | 2.26-2.94 |
| Schizophrenia | 5 967 | 140 | 2.34 | 1.98-2.77 |
| Hypertension | 49 242 | 1 449 | 2.31 | 2.18-2.45 |
| CHD | 9 656 | 348 | 2.26 | 2.02-2.52 |
| Diabetes | 37 198 | 732 | 2.20 | 2.04-2.38 |
| Asthma | 154 462 | 1 275 | 2.14 | 2.02-2.27 |
| Anxiety | 205 306 | 2 545 | 2.06 | 1.97-2.15 |
| Affective | 152 599 | 1 948 | 2.05 | 1.95-2.15 |
| Alcohol problems | 74 148 | 972 | 2.00 | 1.87-2.14 |
| Blindness low | 25 209 | 297 | 1.99 | 1.78-2.24 |
| Migraine | 57 897 | 690 | 1.98 | 1.83-2.14 |
| Painful back | 148 977 | 2 347 | 1.98 | 1.89-2.07 |
| Anorexia bulimia | 22 614 | 173 | 1.92 | 1.65-2.24 |
| Intestine | 12 546 | 323 | 1.91 | 1.70-2.13 |
| IBS | 29 930 | 377 | 1.88 | 1.69-2.08 |
| Arthrosis | 51 157 | 1 186 | 1.87 | 1.76-1.99 |
| Thyroid | 51 053 | 796 | 1.81 | 1.69-1.95 |
| Chronic sinusitis | 10 384 | 154 | 1.75 | 1.49-2.05 |
| Prostate disease | 10 570 | 173 | 1.69 | 1.45-1.97 |
| Multiple sclerosis | 5 637 | 92 | 1.68 | 1.36-2.06 |
| Parkinson’s | 685 | 14 | 1.61 | 0.94-2.72 |
| Dermatitis Eczema | 183 168 | 1 525 | 1.56 | 1.47-1.64 |
| Psoriasis | 35 735 | 445 | 1.49 | 1.36-1.64 |
| Hearing loss | 42 338 | 438 | 1.46 | 1.32-1.61 |
| Glaucoma | 6 348 | 100 | 1.27 | 1.04-1.54 |
| Learning disability | 6 405 | 17 | 1.08 | 0.67-1.74 |
